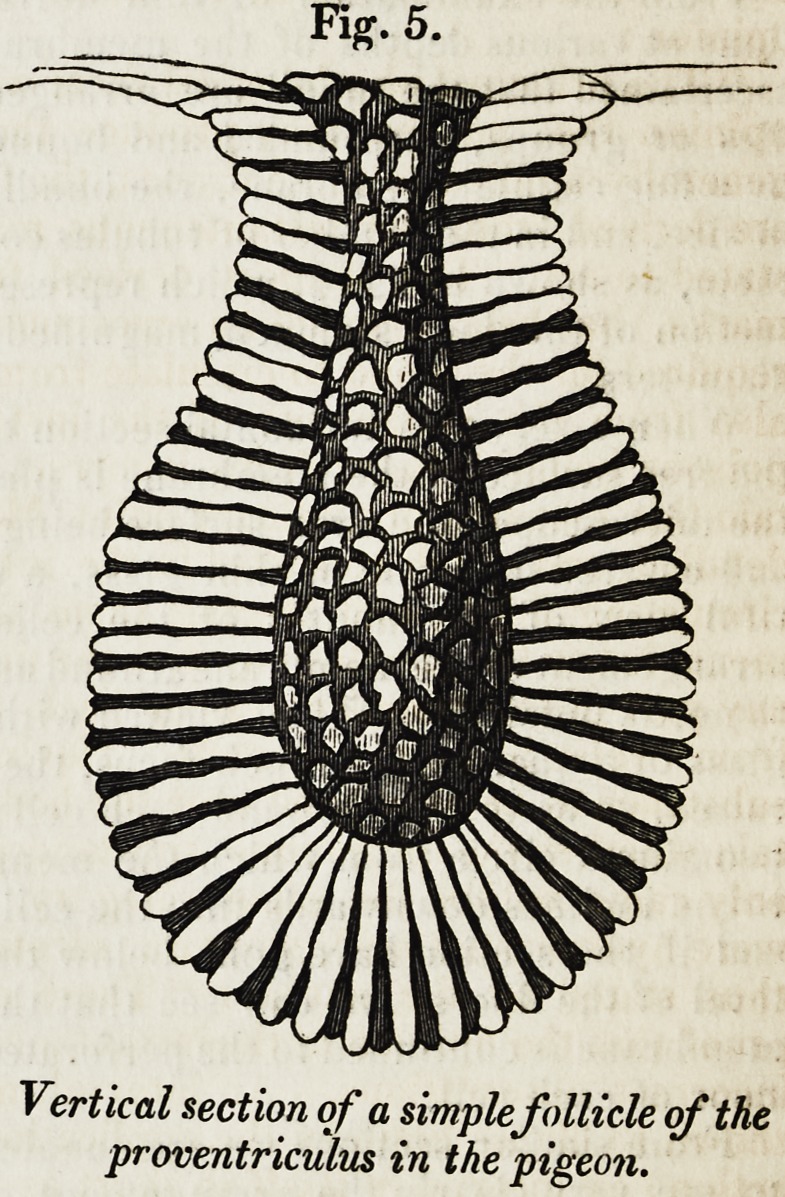# The British Journals: Anatomy and Physiology

**Published:** 1840-04

**Authors:** 


### III. THE BRITISH JOURNALS.

(FOR THE QUARTER ENDING FEBRUARY 29, 1840.)

### ANATOMY AND PHYSIOLOGY.

*On the Structure of the Mucous Membrane of the Stomach.* By R. B. Todd, M.D., F.R.S., Professor of Physiology, King's College, London.

[We regret much that our limits will only allow us to extract the part of this excellent paper which relates to the anatomy; the whole is equally worthy of notice.]

When a portion of the mucous membrane of a true digestive stomach is examined, stretched upon a plane surface under water, we observe it to exhibit a multitude of small cells more or less circular in form. These cells are present over the whole surface, where a thick epithelium visible to the naked eye does not exist, and their presence may be considered to be characteristic of the true digestive surface, as contra-distinguished from that of a simple macerating cavity. When the mucus has been well cleared away, we can see to the floor of each cell, which exhibits from three to five perforations, often rendered distinct by being filled with the white mucous secretion (fig. 1). The cells are separated from each other by partition-like elevations of the membrane, which vary in depth, and sometimes even form pointed processes, mistaken by some anatomists for villi, which they really do resemble when examined on an oblique section. The diameter of the cells is about 1-180th inch to 1-250th inch: it varies, however, in the different regions, and is always largest near the pylorus. Such is the general description of the mucous surface of the stomach of all animals in which I have examined it—in man, the dog, cat, lion, the fourth stomach of ruminants, in the pig, rabbit, horse, and ass; in the simple stomachs of the frog and waternewt, in the stomach of the turtle, and in those of the skate and cod, in the former of which each cell measured 1-360th of an inch.

Fig. 1.

*This figure (from Bischoff) represents the cells seen on the surface of the stomach, with their floors perforated by the orifices of the tubes.*

When the vessels of the stomach have been minutely injected with the size injection coloured red, nothing can be more beautiful than the vascular net-work which is then seen on the surface of the mucous membrane. The margin of each cell is surrounded by a vascular circle, which is joined at various points of its circumference by minute vessels emerging from the substance of the membrane (fig. 3), and all the circles anastomose with each other. I know nothing which more forcibly exhibits the intricacy of the capillary vessels themselves than this vascular net-work on the surface of the gastric mucous membrane.

Although the appearance which I have described is rendered visible by a lens of very low magnifying power, so low as three or four diameters, no trace of it can be seen by the naked eye. The orifices of the so-called gastric glands, which Sir Everard Home states may be seen at the pyloric and cardiac portions of the mucous membrane of the stomach of man and other animals, can correspond to nothing but the cells which I have described; yet it is difficult to imagine, if he really did see these cells at the cardia, how he could have avoided seeing them, similar in arrangement although different in size, all over the mucous surface. Not unfrequently a remarkable series of smaller wrinkles is seen on the pyloric and cardiac portions of the membrane. Three slight and very short fissures radiate from a central depression, and these occur in so great numbers, and at such regular distances from each other, that they are not unlikely to be mistaken for a peculiar structure, nor to be set down as glands, by those who are zealously in search of a distinct series of such organs in connexion with the stomach. I have seen this appearance many times on the human stomach, and always in that of the pig; and I am disposed to think that it is produced by the contraction of the muscular coat, although I am unable to explain exactly the manner in which it is effected.

When thin sections of the mucous membrane, cut vertically to the surface, are placed under the microscope, they are seen to be composed of a number of tubuli closely applied to each other side by side, their blind extremities being in contact with the submucous tissue, and their free extremities opening into the bottom of the cells. In some situations these tubuli are straight and short; in other parts they are longer, and at their blind extremities present an appearance which might arise either from a slight convolution of the tube, or from some irregular dilatations of it in that situation (fig. 2). It very commonly happens that two tubuli coalesce or anastomose at their free extremities, and they will consequently open upon the floor of a cell by a common orifice; and hence it is that a greater number of tubules actually pour their contents into a cell, than would be indicated by the number of openings which pierce its floor. The diameter of the tubuli varies from 1-360th to 1-540th of an inch.

Fig. 2.

*Tubes from the pyloric portion of the stomach, as seen by a vertical section.*

The tubuli are straighter and shorter at the cardiac portion of the stomach, longer and more convoluted or irregular at their blind ends, at the pyloric portions. In a vertical section of an injected specimen, we see the vessels coming up from the submucous tissue, and passing between the tubuli, as in the annexed figure (fig. 3), to terminate in the vascular net-work of the surface.

Fig. 3.

*Vertical section of the mucous membrane, showing the vessels passing to the superficial net-work (from Bischoff).*

From the examination of thin horizontal sections at various depths of the membrane, I have ascertained that the tubuli are arranged in bundles or groups, surrounded and bound together by a fine cellular membrane, the bundles varying in size, and in the number of tubules contained in them, as shown in fig. 4, which represents such a section of the dog's stomach, magnified about 100 diameters.

When a very thin horizontal section taken from the free surface of the membrane is placed under the microscope, the free surface being upwards, and covered with talc or thin glass, a very beautiful view of the mouths of the cells, and the arrangement of the membrane around and between them, is obtained. When viewed with an object glass of a quarter of an inch focus, the membrane surrounding each cell appears raised, so as to form around each cell a prominent circle from which the membrane inclines downwards into the cell; and if the section have gone below the level of the floors, we can see that the membrane is continued to the perforated floor of each cell.

From similar sections we are enabled to see very clearly the arrangement of the epithelium on the surface. I have already stated that the absence of a thick epithelium, visible to the naked eye, is characteristic of the true digestive stomach. An epithelium, however, nevertheless exists, of a very definite arrangement, which is distinctly brought into view by the use of high powers, of a quarter and eighth of an inch focus; and we are indebted to Henle for the first complete demonstration of the existence of an epithelium upon the whole mucous

Fig. 4.

*Transverse section of the tubuli in the dog.*

tract from the mouth to the anus. My observation, however, does not confirm the statements of this anatomist with respect to the gastric mucous membrane: I have never seen the cylindrical form of epithelium in any part of the stomach. The whole surface of the membrane, on the contrary, appears to be, as it were, covered with a pavement of fine polygonal epithelium scales, which under the highest power present an appearance very similar to that of shagreen. The scales not only occupy the space between the cells, but pass over their margins, and are continued down to their floors. The diameter in the scales, in the dog, in the cardiac portion of the stomach, was from 1-3100th to 1-2325th of an inch. These scales resemble very much those of the deep layer of œsophageal epithelium, both in form and dimensions.

The matter contained in the tubuli appears to be of a very different nature from the scales of the epithelium: it is a soft, whitish substance, composed of minute granules, which exhibit no trace of structure even under the highest powers. This matter may be readily obtained by pressure from the tubes, in which it always exists in considerable quantity: it is in every respect the same as the layer of mucus which covers the membrane of the recent stomach.

The structure of the mucous membrane of the proventriculus or true digestive stomach of birds demands a separate description. Here, it will be recollected, the membrane presents a multitude of large follicles, which open into the cavity of the proventriculus. Each follicle may be considered as a little stomach in itself: when a simple follicle is laid open in its long diameter we observe a number of minute orifices on its internal surface, which are those of a series of tubuli arranged side by side, and vertically to its wall. The annexed figure (fig. 5) is a diagram representing a vertical section of one of these follicles in the pigeon: the tubes in this bird are all short and straight, and measure in diameter from 1-540th to 1-720th of an inch. The free surface of the membrane lining the cavity of the proventriculus is covered by a delicate epithelium; the scales of a less distinct character than usual, polygonal, but with one or two of the angles rounded off.

Fig. 5.

*Vertical section of a simple follicle of the proventriculus in the pigeon.*

The follicles of the proventriculus of the ostrich are of the most complex kind: into each follicle a series of smaller and simple follicles pour their secretions, so that one of these compound follicles may be said to represent on a small scale the proventriculus of the pigeon. Each of the smaller follicles has exactly the structure of the simple follicle of the pigeon. The compound follicle, then, consists of an aggregate of simple follicles placed side by side, and vertically to the walls of the larger ones, whilst each simple follicle consists of an aggregate of tubuli as before described. The epithelium is very distinct on the surface of the proventriculus, on which also there are numerous triangular processes not unlike villi.

From the preceding description of the structure of the mucous membrane of the digesting stomach in the vertebrata, we may not improperly designate this membrane as a gland; its constituent tubes being arranged perpendicularly to an extended surface, and pouring their secretions into a number of cells; not, as in other glands, into one or more canals or ducts.

*London Medical Gazette. Dec.* 13, 1839.

*Observations and Experiments on the Mode in which various Poisonous Agents act on the Animal Body.* By Mr. JAMES BLAKE.

THIS is an important contribution to the stock of our knowledge of toxicology, and merits the attention of both physiologists and pathologists. "Of the many opinions (says Mr. Blake) that have been entertained on the manner in which poisons produce their effects, there are only two which it is now necessary to notice, as they express the views of by far the greater number of physiologists of the present day. According to one of these opinions, before a poison can produce any general effects, it is essential that it should be mixed with the blood circulating over the body, and thus brought into contact with the nervous tissue, or, at least, that the poison should, in some manner, be strictly applied to the nervous centres. The other opinion is, that these poisons modify or destroy the functions of the nervous system, by an impression made on the nerves of the part to which they are directly applied, and which, being transmitted to the nervous centres, may destroy these functions independently of any contact of the substance with the nervous tissue generally." It is well known to our readers that the latter opinion has been supposed to derive, if not confirmation, at least strong support from the experiments of Mr. Morgan and Dr. Addison, performed twelve years since. In the present paper, Mr. Blake endeavours to show the inconclusiveness of these experiments, and to establish the reverse opinion:

"The principal physiological facts which have been supposed to support the opinion that poisons may produce their effects on the system, without being generally applied to those tissues, the function of which they appear to destroy, are derived from the instantaneous manner in which some poisons have been stated to act. The support derived from this fact, however, (Mr. Blake remarks,) has been founded on erroneous views which have been taken of the time required for the blood to circulate from one part of the system to another; and also from the statements of the instantaneous action of the more powerful poisons being deduced from incorrect observations." And, in order to ascertain this essential preliminary point, Mr. B. instituted several experiments here detailed, and which consisted in the introduction of certain substances into the circulating fluids, and tracing them from one part to another. These experiments led to the conclusion, that the time required for a substance to pass from any part of the vascular system back to the same part again (in dogs) varies from twelve to twenty seconds. Having thus obtained the time requisite for a substance to be circulated over the body, the experimenter proceeded to ascertain whether the poisonous substances alleged to act on the nervous system only came within the same category. The substances used for this purpose were hydrocyanic acid, woorara, nicotine, conia, and strychnia. The result is thus stated by the author: "These experiments, I think, furnish sufficient proof that the opinion of the instantaneous action of poisons must be founded on incorrect observations. An interval, never less than twelve seconds, has been shown to elapse between the application of a poison and the first symptom of its action; an interval in itself so short as might almost justify its being neglected, particularly in the present state of opinion of physiologists on the rapidity with which the organic processes are carried on; but which becomes of importance when compared with the time actually occupied for the performance of these processes, as it is quite sufficient for a poison to be brought into general contact with those tissues it affects. This being the case, it is unphilosophical to suppose that these effects on the nervous centres are owing to an impression communicated to them by the nerves, and not the result of the direct application of the poison to them." These conclusions are still further strengthened by experiments, which show "that the nearer to the nervous centres is the part of the vascular system into which the poison is introduced, the more rapid is its action."

Before concluding his paper, Mr. Blake briefly examines the experiments instituted by Mr. Morgan and Dr. Addison, and then states the general conclusions derived from his own, as follows:

1. That the time required* by a substance to permeate the capillary vessels may be considered as inappreciable.

2. That the interval elapsing between the absorption of a substance by the capillaries and its general diffusion through the body may not exceed nine seconds.

3. That an interval always more than nine seconds elapses between the introduction of a poison into the capillaries or veins and the appearance of its first effects.

4. That, if a poison be introduced into a part of the vascular system nearer the brain, its effects are produced more rapidly.

5. That the contact of a poison with a large surface of the body is not sufficient to give rise to general symptoms, as long as its general diffusion through the body is prevented.

*Edinburgh Journal. Jan.* 1, 1840.

---

*Observations on Corpora Lutea.* By ROBERT PATERSON, M.D., Physician to the Leith Dispensary.

[THIS is an elaborate anatomical history of the appearances in the ovaries which are termed *corpora lutea.* The present is only the first part of the essay, and contains—1st, The appearances presented by the true corpus luteum in its early stages, its anatomical position, and the time and mode of its disappearance. 2d, The different kinds of false corpora lutea. The much-debated question, whether or not the true corpus luteum ever occurs in the human subject, or any of the lower animals, without impregnation having taken place, is left for future consideration. The following are stated by Dr. Paterson to be the different sources whence *false* corpora lutea may arise, and the marks which distinguish them from the *true* :]

False corpora lutea may arise :

*First,* From the bursting and subsequent filling of a vesicle with blood, as in menstruation.

*Second,* From partial effusion of blood into a vesicle, either with or without rupture of it.

*Third,* By reabsorption of the fluid of a morbidly enlarged Graafian vesicle, giving rise to a puckered cyst.

*Fourth,* From effusion of blood into the tissue of the ovary, the apoplexy of that organ.

*Fifth,* Tubercular deposits.

*Sixth,* Cysts filled with yellow fatty matter.

These are to be distinguished from the true corpus luteum by the following marks:

They in general have an irregular form.

They want the central cavity lined with a distinct membrane, or the central puckered cicatrix.

They have no concentric radii.

They are frequently numerous in both ovaries.

*Edinburgh Journal. Jan.* 1, 1840.

* In regard to the statement as to the time required for substances to be diffused through the body, I would observe that all my experiments have been performed on full-grown dogs. It is probable that these observations might require to be slightly modified in applying them to other animals. It is also evident that a difference must exist in the time occupied by a substance in reaching the capillaries in different parts of the body. The time here given, or nine seconds, is that in which I conclude a substance may be applied over the greater part of the body, more particularly to the central parts of the nervous system. It would appear that seven seconds may suffice for its being conveyed to the capillary terminations of the coronary arteries. *Ed. Journal.*

*Four Cases of Aneurism of the Arch of the Aorta, and a Case of Diaphragmatic Hernia.* By JOHN REID, M.D., F.R.C.P.E., Lecturer on Physiology.

DR. REID informs us that, beside the cases here detailed, he has had occasion, during the last eighteen months, to inspect in the Royal Infirmary five other cases of fatal thoracic aneurism. Of these—in one, the aneurism was placed upon the middle part of the thoracic portion of the aorta, and burst into the œsophagus; a second was placed upon the lower part of the thoracic and upper part of the abdominal aorta, and burst into the left cavity of the pleura; in a third, the aneurism was placed upon the transverse portion of the arch of the aorta, had destroyed by its pressure the continuity of the left recurrent nerve, and produced sudden suffocation by its effects upon the movements of the muscles attached to the arytenoid cartilages of the larynx; in a fourth, the aneurism occupied the upper part of the descending portion of the arch, and proved fatal by its pressure upon the trachea and left recurrent nerve; in the fifth, the aneurism was placed upon the trunk of the arteria innominata at its origin, had acquired an immense size, extending upwards as high as the thyroid cartilage of the larynx, and produced absorption of the upper part of the sternum, and of the inner end of the right clavicle. The skin had become livid at two places over the surface of the tumour, the blood had even begun to ooze out, but he died before any external rupture had occurred.

The four cases given in full are, from the rarity of their termination, and the precise manner in which they are detailed, valuable contributions to the morbid anatomy of aneurism of the arch of the aorta. The first case terminated fatally *by opening into the right auricle;* the second, *by bursting into the pulmonary artery;* the third, *by communicating with the pulmonary artery*, in the position of the *ductus arteriosus*, but not, as Dr. Reid believes, from this duct continuing pervious; the fourth, *by bursting externally.*

*Edinburgh Journal. Jan.* 1, 1840.

## Figures and Tables

**Fig. 1. f1:**
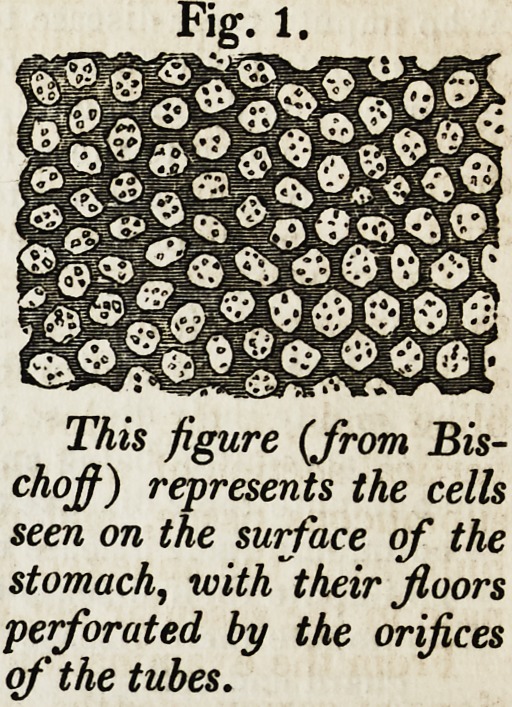


**Fig. 2. f2:**
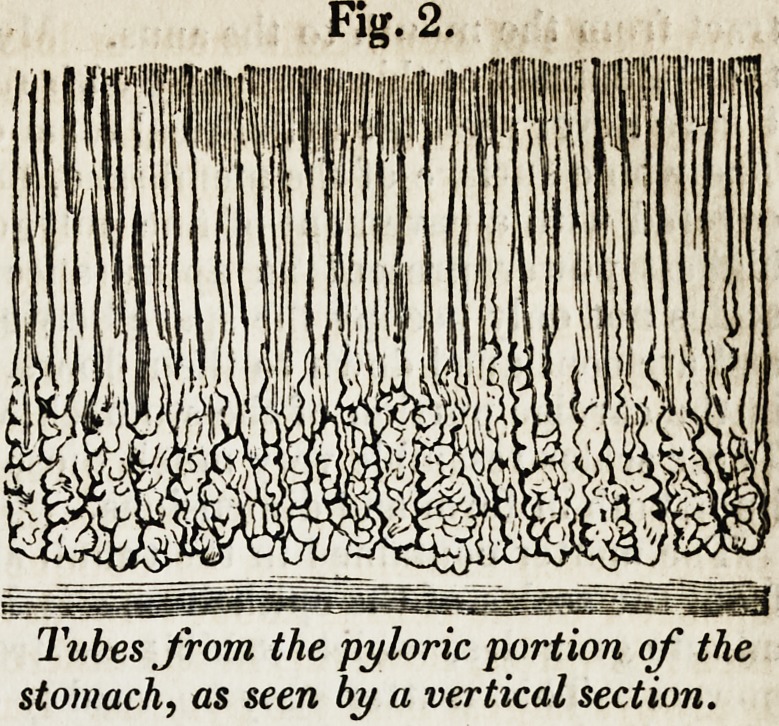


**Fig. 3. f3:**
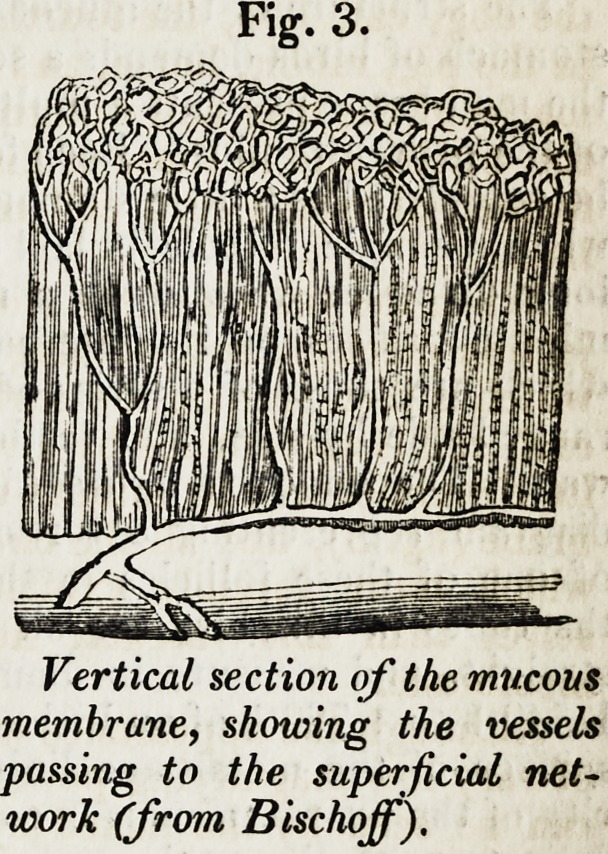


**Fig. 4. f4:**
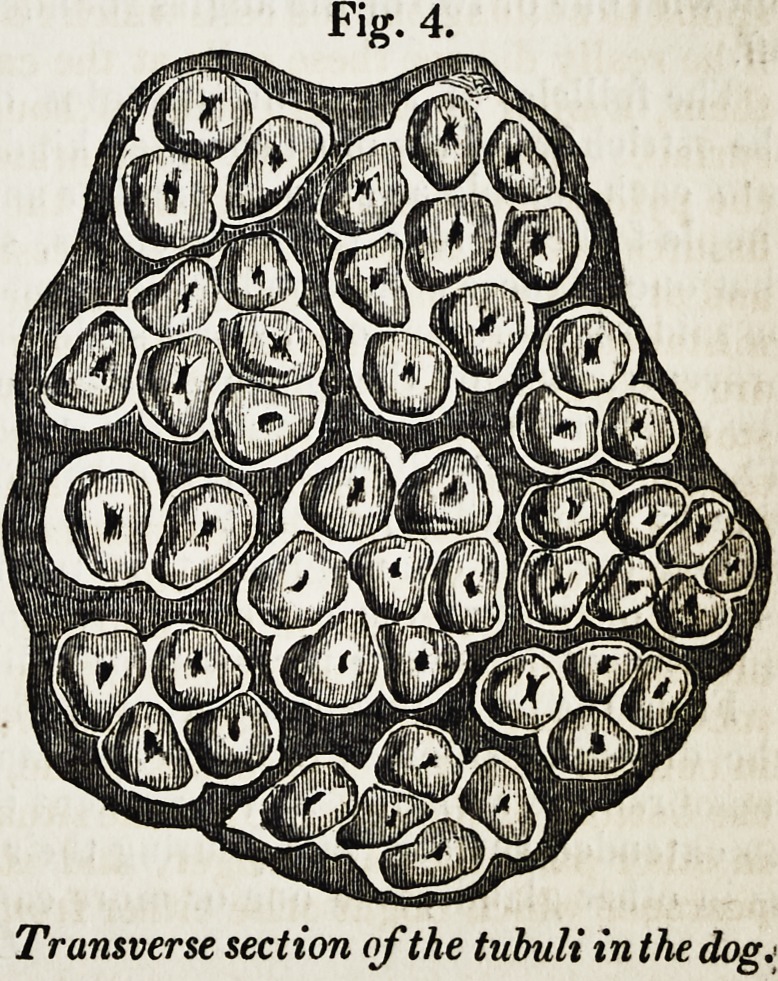


**Fig. 5. f5:**